# Understanding lymphatic filariasis risk in endemic regions: Associations with preventive behaviours, environmental factors, and other mosquito-borne diseases

**DOI:** 10.1016/j.onehlt.2026.101614

**Published:** 2026-10-15

**Authors:** Yoerdy Agusmal Saputra, Rohana Uly Pradita Siregar

**Affiliations:** a Department of Environmental Health, Faculty of Public Health, Universitas Sriwijaya, Indralaya 30862, Indonesia; b Department of Public Health and Epidemiology, Faculty of Medicine, Universitas Pelita Harapan, Tangerang 15811, Indonesia

**Keywords:** Lymphatic filariasis, Mass drug administration, Mosquito-borne diseases, One health, Sanitation

## Abstract

Lymphatic filariasis (LF) remains a major public health challenge in Indonesia, where transmission persists despite more than a decade of mass drug administration (MDA). Using data from the nationally representative Indonesia Health Survey (SKI 2023) (*n* = 520,505), we examined the demographic, behavioural, environmental, and other mosquito-borne disease associations of self-reported LF in Indonesia. We conducted a cross-sectional, weighted analysis of SKI 2023 with multivariable logistic regression and rural/urban stratified models to identify factors independently associated with LF. Overall self-reported LF prevalence was 1.9%. After adjustment, children aged 0–17 years had higher odds of LF (aOR = 1.36; *p* < 0.001), and females had slightly increased odds (aOR = 1.07; *p* = 0.001). Skipping preventive drugs under health worker supervision was strongly associated with LF (aOR = 2.49, *p* < 0.001). Use of pit or open-hole latrines was independently associated with increased odds (aOR = 1.15; *p* < 0.001). Paradoxically, not sleeping under insecticide-treated nets (≤3 years or > 3 years) was associated with lower odds of LF (aOR = 0.91; *p* = 0.044 and aOR = 0.83; *p* < 0.001, respectively). Co-occurrence with malaria and dengue was observed in bivariate analyses (ORs 2.63; 4.31), suggesting overlapping vector ecologies. Persistent, heterogeneous LF in Indonesia is linked to gaps in supervised MDA, inadequate sanitation, and overlap with other mosquito-borne diseases. Strengthening directly observed MDA, integrating WaSH and locally tailored vector management, and enhancing entomological and parasitological surveillance are critical for evidence-based, multisectoral One Health strategies to accelerate national LF elimination.

## Highlights

• Skipping supervised MDA doubles odds of LF (aOR = 2.49).
• Use of pit/open-hole latrines linked to higher LF odds (aOR = 1.15).
• Malaria and dengue history strongly co-occur with LF infection.

## 1 Introduction

Lymphatic filariasis (LF) is a mosquito-transmitted parasitic disease that causes disabling conditions such as lymphoedema and hydrocele [1], [2] and imposes substantial socioeconomic burdens; affected individuals often lose workdays [3], [4], [5], incur high medical costs [4], [5], and experience stigma [4]. These chronic impacts make LF a priority for tropical disease elimination programmes that integrate public health interventions with broader development goals.

Globally, over 120 million are infected with LF, and tens of millions have disabling disease; hundreds of millions remain at risk [1], [6]. In Southeast Asia approximately 63% of the region's 1.34 billion people are at risk [7], [8]. In Indonesia, 5601 chronic cases were reported, and 236 of 514 districts/cities remained endemic in 2023–2024, indicating nearly 46% of administrative units continued transmission despite >10 years of mass drug administration [9]. Progress is heterogeneous: 210 endemic districts/cities completed a five-year MDA cycle and entered post-elimination surveillance, while 26 (11.0%) still conduct MDA. The burden concentrates in eastern provinces; Papua reported 1023 cases (19%), and East Nusa Tenggara 955 cases (17%) [1], [8], [9].

Indonesia is unique epidemiologically because it harbours all three human filarial species (*Wuchereria bancrofti*, *Brugia malayi*, and *Brugia timori*), each associated with different vector groups and transmission ecologies [10], [11]. Vector biting behaviour varies by genus and species: major nocturnal vectors (e.g., *Culex*, *Mansonia*, and *Anopheles*) contrast with diurnally active *Aedes* in some settings [12], [13], [14], [15]. Such variation affects the likely impact of personal protective measures—for example, the use of bed nets at night is more likely to reduce exposure if the dominant vectors are nocturnal but will be less effective against day-biting vectors [16], [17].

The extensive demographic and socio-economic context of Indonesia, along with the geographical variation between coastal settlements, wetlands, and mountains, creates a diverse ecosystem that influences the distribution patterns of vector mosquitoes [1], [8], [10], [18]. The increase in urbanisation in Indonesia has changed settlement patterns and infrastructure, often resulting in population concentration in urban/peri-urban areas accompanied by informal settlements and inadequate sanitation [10], [19]. This phenomenon alters habitat patterns that affect vector distribution: *Culex* is often associated with poor urban and semi-urban sanitation, *Mansonia* is more common in swamp or coastal areas, *Anopheles* is primarily found in rural areas, and *Aedes* mosquitoes are mainly found in island regions [1], [10]. The higher national poverty rate in rural areas also affects the social determinants of health—access to services, housing, and sanitation—which impacts exposure risk and access to healthcare services [18], [20].

Current elimination strategies combine repeated MDA to reduce the human parasite reservoir with vector control and morbidity management to prevent disability [16], [21]. Programme effectiveness, however, depends on multiple interacting determinants: MDA coverage and individual compliance [22], sociodemographic factors [23], personal preventive behaviours (net and repellent use) [1], [24], [25], [26], environmental sanitation [27], [28], [29], and interactions with other mosquito-borne diseases [30], [31]. Heterogeneity in these determinants, together with methodological limitations in prior studies (measurement variability, residual confounding, and contextual differences), constrains our ability to quantify the relative contribution of each factor to ongoing transmission.

To address these gaps, research designed to quantify the association between behavioural determinants, sanitation conditions, and co-infections with LF transmission indicators is urgently needed. The goal of these studies is to provide more detailed empirical evidence so that policymakers and programme designers can optimise the combination of interventions—integrating MDA, mosquito bite prevention behaviours (use of bed nets, mosquito repellents, and other mosquito control products), and sanitation improvements (presence and location of household defecation facilities, toilet type, and faecal waste disposal methods)—to accelerate the achievement of elimination targets in endemic areas of Indonesia.

## 2 Methods

### 2.1 Study design

This study used secondary data from the 2023 Indonesia Health Survey (Survei Kesehatan Indonesia/SKI 2023), a nationally representative survey conducted using a multistage stratified cluster sampling design across all provinces of Indonesia. The survey collected information on demographic characteristics, health conditions, preventive behaviours, and environmental sanitation at the household and individual levels. Sampling weights provided by SKI 2023 were applied in all analyses to account for the complex survey design and to ensure national representativeness. The analytic dataset included 520,505 respondents with complete information on filariasis status and selected covariates.

### 2.2 Variable

The outcome variable was self-reported lymphatic filariasis diagnosed by a health professional, as recorded in the SKI 2023 questionnaire. The survey captures chronic clinical manifestations consistent with LF, including lymphoedema and hydrocele. These manifestations represent long-term sequelae of prior infection and do not necessarily indicate active parasitological infection at the time of survey. The survey did not include parasitological confirmation, such as circulating filarial antigen testing, microfilaria detection, or molecular diagnostics. Filariasis status was coded as a binary variable (no filariasis vs. filariasis). Because SKI 2023 is a population-based survey conducted across multiple provinces and districts, the dataset reflects the heterogeneous geographic distribution of filariasis cases across Indonesia, although species-specific infection and parasite reservoir information were not available in the survey.

Independent variables included demographic characteristics, preventive drug use, mosquito-bite prevention behaviours, environmental sanitation indicators, and co-occurrence with other mosquito-borne diseases. Demographic variables included age group, gender, and area of residence (urban or rural). Preventive drug variables included the receipt of filariasis preventive drugs and whether drug intake was supervised by health workers. Mosquito-bite prevention variables included sleeping under bed nets, sleeping under insecticide-treated bed nets, use of mosquito repellents, and household mosquito-control products. Environmental sanitation variables included availability of defecation facilities, location of defecation facilities, type of toilet used, and faecal waste disposal method. The analysis also included self-reported malaria and dengue to examine co-occurrence with other mosquito-borne diseases.

### 2.3 Statistical analysis

Descriptive statistics were used to summarise participant characteristics and filariasis prevalence. Bivariate associations between independent variables and filariasis were assessed using odds ratios (ORs) with 95% confidence intervals (CIs). A multivariable logistic regression model was used to estimate adjusted odds ratios for factors associated with filariasis after controlling for demographic characteristics, preventive drug use, mosquito-bite prevention behaviours, environmental sanitation variables, and co-occurrence with malaria and dengue. To examine variation across settings, stratified multivariable models were estimated separately for rural and urban populations. Statistical significance was determined at *p* < 0.05. All analyses were performed using SPSS version 30.0 (IBM Corp., Armonk, NY, USA).

### 2.4 Ethical consideration

This study was approved by the Health Research Ethics Committee, Faculty of Public Health, Universitas Sriwijaya, No. 680/UN9.FKM/TU.KKE/2025.

## 3 Results

A total of 520,505 respondents were included in the analysis, of whom 9784 (1.9%) reported filariasis and 510,721 (98.1%) did not. Fig. 1 shows the distribution of filariasis cases across socioeconomic groups. The highest number of filariasis cases was observed in the bottom socioeconomic group (*n* = 229), followed by the lower-middle (*n* = 191), middle (*n* = 187), upper-middle (*n* = 186), and top socioeconomic groups (*n* = 183). The number of respondents without filariasis was relatively similar across socioeconomic categories. Overall, filariasis cases were present across all socioeconomic levels, with slightly higher counts observed in lower socioeconomic groups.

**Fig. 1:**
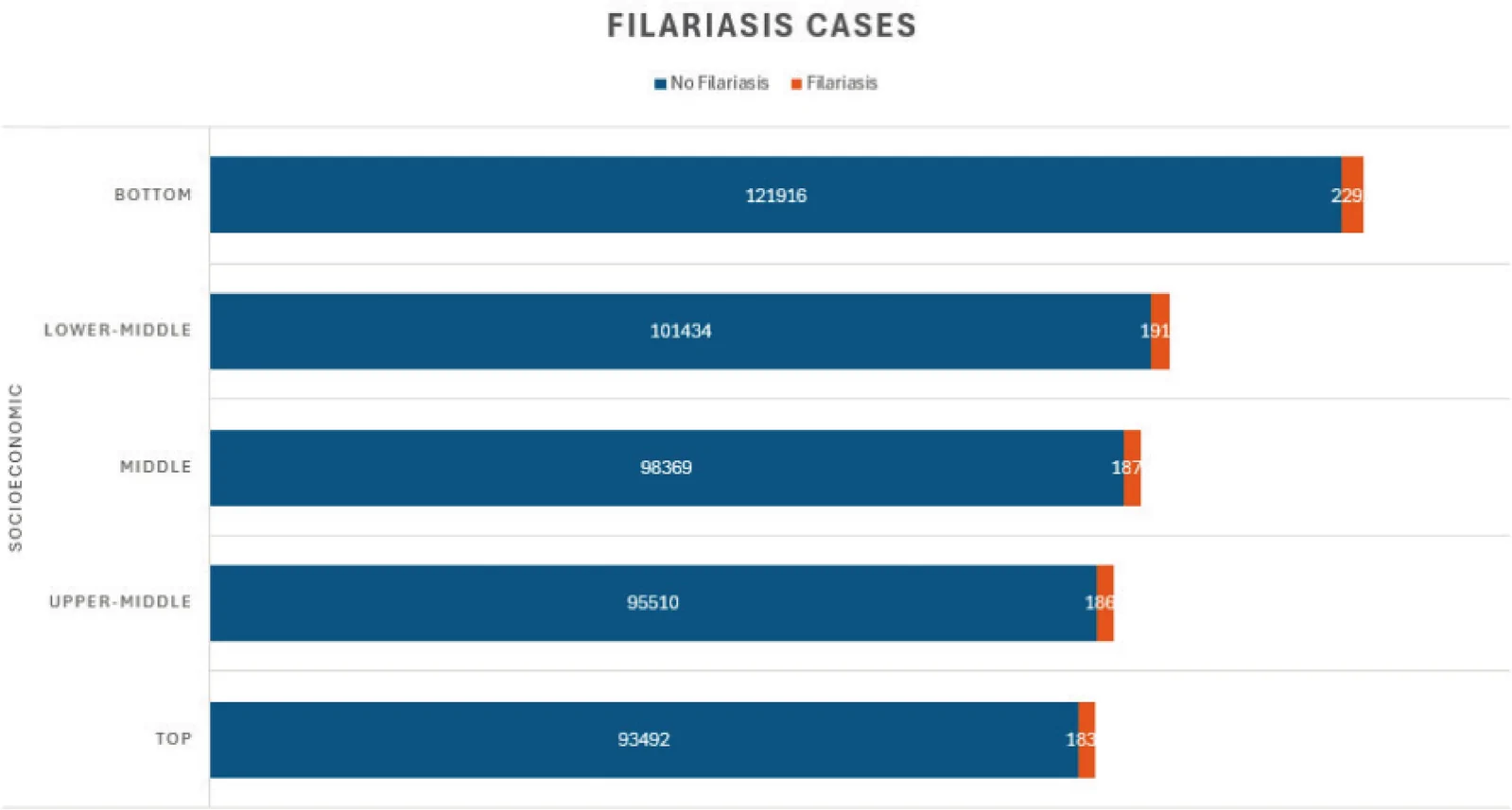
Association between socioeconomic status and LF cases.

As shown in Table 1, compared with respondents aged ≥60 years, children aged 0–17 years had higher odds of filariasis (OR = 1.391, 95% CI: 1.291–1.500, *p* < 0.001), while respondents aged 18–59 years did not differ significantly (OR = 1.040, 95% CI: 0.967–1.119, *p* = 0.290). Females had slightly higher odds of filariasis compared with males (OR = 1.047, 95% CI: 1.006–1.090, *p* = 0.026). Respondents living in urban areas had slightly higher odds of filariasis compared with those living in rural areas (OR = 1.051, 95% CI: 1.010–1.094, *p* = 0.015).

**Table 1:** Characteristics of participants.

	No filariasis	Filariasis	Total	Odds ratio	p-value
	(*n* = 510,721)	(*n* = 9784)	(*n* = 520,505)	(95% CI)	
Age					
≥ 60 years old	51,020 (10.0)	851 (8.7)	51,871 (10.0)	ref	ref
18–59 years old	295,841 (57.9)	5131 (52.4)	300,972 (57.8)	1.040 (0.967–1.119)	0.290
0–17 years old	163,860 (32.1)	3802 (38.9)	167,662 (32.2)	1.391 (1.291–1.500)	< 0.001

Gender					
Male	240,287 (47.0)	4492 (45.9)	244,779 (47.0)	ref	ref
Female	270,434 (53.0)	5292 (54.1)	275,726 (53.0)	1.047 (1.006–1.090)	0.026

Area					
Rural	276,470 (54.1)	5175 (52.9)	281,645 (54.1)	ref	ref
Urban	234,251 (45.9)	4609 (47.1)	238,860 (45.9)	1.051 (1.010–1.094)	0.015

Preventive drug use, mosquito-bite prevention behaviours, and environmental sanitation factors are presented in Table 2. Respondents who did not receive filariasis preventive drugs had higher filariasis prevalence (2.1%) compared with those who received preventive drugs (1.2%), corresponding to higher odds of filariasis (OR = 1.738, 95% CI: 1.649–1.833, *p* < 0.001). Similarly, respondents who did not take preventive drugs under health worker supervision had higher prevalence (2.1%) compared with those who took the drugs (0.9%), with substantially higher odds of filariasis (OR = 2.327, 95% CI: 2.178–2.487, *p* < 0.001).

**Table 2:** Preventive behaviours and environmental factors.

	No filariasis	Filariasis	Total	Odds ratio	p-value
	(n = 510,721)	(n = 9784)	(n = 520,505)	(95% CI)	
Preventive drug use					
Received filariasis preventive drug					
Yes	134,852 (98.8)	1674 (1.2)	136,526 (100.0)	ref	ref
No	375,928 (97.9)	8112 (2.1)	384,040 (100.0)	1.738 (1.649–1.833)	<0.001
Took drug under health worker supervision					
Yes, complete and partial	104,609 (99.1)	975 (0.9)	105,584 (100.0)	ref	ref
No	406,171 (97.9)	8811 (2.1)	414,982 (100.0)	2.327 (2.178–2.487)	<0.001

Mosquito bite prevention					
Sleeps under a non-insecticide-treated bed net					
Yes	145,659 (98.1)	2746 (1.9)	148,405 (100.0)	ref	ref
No	365,062 (98.1)	7038 (1.9)	372,100 (100.0)	1.023 (0.978–1.069)	0.330
Sleeps under an insecticide-treated bed net that is ≤3 years old					
Yes	32,712 (98.0)	683 (2.0)	33,395 (100.0)	ref	ref
No	478,009 (98.1)	9101 (1.9)	487,110 (100.0)	0.912 (0.843–0.986)	0.023
Sleeps under an insecticide-treated bed net that is >3 years old					
Yes	26,643 (97.8)	596 (2.2)	27,239 (100.0)	ref	ref
No	484,078 (98.1)	9188 (1.9)	493,266 (100.0)	0.848 (0.780–0.923)	< 0.001
Uses mosquito repellents or other bite prevention substances					
Yes	54,491 (97.9)	1155 (2.1)	55,646 (100.0)	ref	ref
No	456,230 (98.1)	8629 (1.9)	464,859 (100.0)	0.892 (0.839–0.949)	< 0.001
Uses mosquito control products (coil/spray/electric)					
Yes	275,115 (98.2)	5011 (1.8)	280,126 (100.0)	ref	ref
No	235,606 (98.0)	4773 (2.0)	240,425 (100.0)	1.112 (1.069–1.158)	< 0.001

Environmental sanitation					
The household has a defecation facility					
Yes	489,776 (98.1)	9360 (1.9)	499,136 (100.0)	ref	ref
No	20,945 (98.0)	424 (2.0)	21,369 (100.0)	1.059 (0.960–1.169)	0.262
Defecation facility location					
Inside the house	469,528 (98.1)	8932 (1.9)	478,460 (100.0)	ref	ref
Outside the house	41,193 (98.0)	852 (2.0)	42,045 (100.0)	1.087 (1.013–1.167)	0.021
Type of toilet used					
Goose-neck toilet	454,186 (98.2)	8555 (1.8)	462,741 (100.0)	ref	ref
Pit latrine/open hole	56,535 (97.9)	1229 (2.1)	57,764 (100.0)	1.154 (1.086–1.226)	< 0.001
Faecal waste disposal method					
Septic tanks or sewage systems	423,382 (98.1)	8183 (1.9)	431,565 (100.0)	ref	ref
Pit, soil, river, pond, or others	87,339 (98.2)	1601 (1.8)	88,940 (100.0)	0.948 (0.899–1.001)	0.057

Sleeping under a non-insecticide-treated bed net was not significantly associated with filariasis (OR = 1.023, *p* = 0.330). Sleeping under insecticide-treated bed nets ≤3 years old and > 3 years old showed slightly higher filariasis prevalence among users compared with non-users, with statistically significant associations (OR = 0.912, *p* = 0.023; OR = 0.848, *p* < 0.001, respectively). Use of mosquito repellents was also associated with slightly higher prevalence among users compared with non-users (OR = 0.892, *p* < 0.001). Households not using mosquito control products had higher odds of filariasis (OR = 1.112, *p* < 0.001). Across several mosquito-bite prevention variables, higher prevalence was observed among respondents reporting preventive behaviours, while household mosquito-control products showed the opposite pattern.

For environmental sanitation variables, the presence of a household defecation facility was not significantly associated with filariasis (OR = 1.059, *p* = 0.262). Households with defecation facilities located outside the house had slightly higher odds of filariasis (OR = 1.087, *p* = 0.021). Use of pit latrines or open holes was associated with higher odds of filariasis compared with goose-neck toilets (OR = 1.154, *p* < 0.001). The faecal waste disposal method was not statistically significant (*p* = 0.057).

Adjusted associations are presented in Table 3. Children aged 0–17 years remained at higher odds of filariasis in the total model (aOR = 1.360, *p* < 0.001), including in rural and urban stratified models. Females also had slightly higher odds in the total model (aOR = 1.071, *p* = 0.001). Not taking preventive drugs under health worker supervision remained strongly associated with filariasis (aOR = 2.485, *p* < 0.001) in both rural and urban models. Not sleeping under insecticide-treated bed nets (≤3 years or > 3 years) remained associated with lower odds of filariasis in the total model, particularly in rural areas. Not using mosquito repellents also showed lower odds in the adjusted model, especially in rural areas. Households not using mosquito control products had higher odds of filariasis in the total model and in both rural and urban stratified models. Use of pit latrines or open holes remained associated with higher odds of filariasis in adjusted analyses, while defecation facility location was not significant. Several associations differed between rural and urban stratified models, indicating variation in the distribution of risk factors across settings.

**Table 3:** Multivariate analysis of characteristics, preventive behaviours and environmental factors.

	Total		Rural		Urban	
	Odds ratio (95% CI)	p-value	Odds ratio (95% CI)	p-value	Odds ratio (95% CI)	p-value
Age						
≥ 60 years old	ref	ref	ref	ref	ref	ref
18–59 years old	1.063 (0.988–1.143)	0.104	1.191 (1.072–1.323)	0.001	0.948 (0.856–1.050)	0.305
0–17 years old	1.360 (1.262–1.466)	<0.001	1.567 (1.408–1.745)	<0.001	1.175 (1.058–1.306)	0.003
Gender						
Male	ref	ref	ref	ref	ref	ref
Female	1.071 (1.028–1.114)	0.001	1.080 (1.022–1.142)	0.006	1.059 (0.999–1.123)	0.055

Preventive drug use						
Received filariasis preventive drug						
Yes	ref	ref	ref	ref	ref	ref
No	0.921 (0.851–0.996)	0.038	0.906 (0.815–1.006)	0.064	0.934 (0.831–1.051)	0.256
Took drug under health worker supervision						
Yes, complete and partial	ref	ref	ref	ref	ref	ref
No	2.485 (2.253–2.742)	<0.001	2.616 (2.300–2.975)	<0.001	2.291 (1.967–2.669)	<0.001

Mosquito bite prevention						
Sleeps under an insecticide-treated bed net that is ≤3 years old						
Yes	ref	ref	ref	ref	ref	ref
No	0.919 (0.847–0.998)	0.044	0.886 (0.808–0.972)	0.011	1.045 (0.867–1.260)	0.646
Sleeps under an insecticide-treated bed net that is >3 years old						
Yes	ref	ref	ref	ref	ref	ref
No	0.827 (0.759–0.902)	<0.001	0.797 (0.722–0.879)	<0.001	0.930 (0.771–1.122)	0.448
Uses mosquito repellents or other bite prevention substances						
Yes	ref	ref	ref	ref	ref	ref
No	0.896 (0.842–0.953)	0.001	0.745 (0.677–0.819)	<0.001	1.018 (0.937–1.106)	0.675
Uses mosquito control products (coil/spray/electric)						
Yes	ref	ref	ref	ref	ref	ref
No	1.093 (1.049–1.139)	<0.001	1.136 (1.073–1.203)	<0.001	1.067 (1.006–1.133)	0.032

Environmental sanitation						
Defecation facility location						
Inside the house	ref	ref	ref	ref	ref	ref
Outside the house	0.966 (0.887–1.053)	0.430	0.934 (0.845–1.033)	0.186	1.108 (0.937–1.310)	0.229
Type of toilet used						
Goose-neck toilet	ref	ref	ref	ref	ref	ref
Pit latrine/open hole	1.148 (1.067–1.235)	<0.001	1.147 (1.048–1.254)	0.003	1.157 (1.021–1.312)	0.023

The co-occurrence of filariasis with malaria and dengue is shown in Table 4. Filariasis prevalence was higher among respondents reporting malaria (4.7%) compared with those without malaria (1.8%) (OR = 2.631, 95% CI: 2.343–2.955, *p* < 0.001). Similarly, respondents reporting dengue had substantially higher filariasis prevalence (7.5%) compared with those without dengue (1.8%) (OR = 4.313, 95% CI: 3.744–4.930, *p* < 0.001).

**Table 4:** Co-occurrence of LF with malaria and dengue.

	No filariasis	Filariasis	Total	Odds ratio	p-value
	(n = 510,721)	(n = 9784)	(n = 520,505)	(95% CI)	
Malaria					
No	504,468 (98.2)	9475 (1.8)	513,943 (100.0)	ref	ref
Yes	6253 (95.3)	309 (4.7)	6562 (100.0)	2.631 (2.343–2.955)	< 0.001

Dengue					
No	507,786 (98.2)	9546 (1.8)	517,332 (100.0)	ref	ref
Yes	2935 (92.5)	238 (7.5)	3173 (100.0)	4.313 (3.744–4.930)	< 0.001

## 4 Discussion

The present nationally representative analysis (*n* = 520,505; filariasis prevalence 1.9%; 9784 cases) identifies a pattern of demographic, preventive behavioural, and environmental associations with self-reported chronic clinical filariasis consistent with persistent heterogeneous transmission across the country (see Table 1, Table 2, Table 3). Children (0–17 years) had higher adjusted odds of filariasis than the oldest age group (aOR 1.36), females had slightly higher odds than males (aOR 1.07), urban residence showed a small positive association in crude analyses, and failure to take preventive drugs under health-worker supervision was strongly associated with filariasis (aOR 2.49). Environmental sanitation—specifically use of pit latrines/open holes—was associated with increased odds of filariasis in adjusted models (aOR 1.15). Finally, self-reported malaria and dengue co-occurrence were strongly associated with higher filariasis prevalence in bivariate analysis (OR 2.63; 4.31).

Receiving and consuming LF preventive medication—whether fully or partially—under the supervision of health officers is associated with a reduced risk of LF. A field study in Papua reported that adherence to treatment during the MDA campaign, particularly the consumption of the full recommended dose, is significantly related to a decrease in LF incidence [32]. Additionally, research in Tanzania indicates that the presence of groups of individuals who consistently do not comply can delay elimination efforts [33]. This phenomenon likely reflects that individuals who are not directly supervised are more prone to missing doses or being systematically overlooked by campaigns, allowing hidden parasite reservoirs to persist [34]. These findings underscore the central role of medication adherence in elimination strategies. Supervised mass drug administration ensures that each recipient swallows the full dose and reduces systematic non-compliance or forgetfulness. The results of our study align with evidence that direct observation is a crucial pillar in improving MDA compliance [35]. Thus, strengthening the implementation of supervised MDA—such as through the involvement of community volunteers and local healthcare workers for social mobilisation and direct observation—is a key strategy to improve actual drug intake and programme performance [36].

Another important finding was that the type of household sanitation had an effect on LF risk. The use of pit latrines or open holes was associated with higher LF odds (aOR = 1.15). This finding is in line with a meta-analysis showing that sanitation plays a significant role in LF transmission [37]. Pit latrines, outdoor and semi-permanent latrines, and open drainage systems create humid, stagnant microhabitats that serve as important breeding sites for *Culex* mosquitoes—the principal vectors of LF in many Indonesian settings, specifically in urban ones. [10], [38], [39], [40]. Several studies indicate that unprotected or poorly ventilated pit latrines create damp and stagnant microhabitats that support the development of *Culex* larvae, with greater *Culex* abundance reported in such facilities compared to well-ventilated pit latrines [41], [42], [43], [44], [45]. In accordance with the integrated WaSH–LF control strategy, improving sanitation at the site (e.g., safe and ventilated systems) and closing latrine openings or installing pipe extensions can reduce mosquito access and LF risk [42].

We observed a significant association between reported ITN use—stratified by net age (≤3 years or > 3 years)—and higher LF prevalence, particularly in rural settings. The apparently paradoxical finding that some personal protective measures (e.g., bed nets, repellents) are not consistently linked to lower odds of infection merits cautious interpretation. Three non-exclusive explanations are plausible. Firstly, reverse causality or confounding by indication may occur when net and repellent use is greater in communities or households with high vector density or prior disease experience, producing a non-causal positive association in cross-sectional data. Secondly, targeted bed-net campaigns for malaria do not always coincide spatially with filariasis hotspots; furthermore, reduced risk perception during the dry season—when malaria and LF vectors and seasonal patterns often overlap—can lead to prolonged periods without ITN use, and given LF's chronic nature, this gap may increase disease risk [46]. Thirdly, vector bionomic heterogeneity across the nation (e.g., nocturnal *Culex*/*Mansonia*/*Anopheles* versus diurnal *Aedes* in some locations) means sleeping under an ITN will only reduce exposure where nocturnal vectors predominate; contributions from diurnal vectors limit ITN protection [14], [47]. Entomological work from Kenya exemplifies this complexity: Long-lasting insecticidal net (LLIN) scale-up suppressed indoor biting and resting of anthropophilic vectors but coincided with increased outdoor transmission and higher LF prevalence in outdoor-resting mosquitoes [48]. Finally, insecticide resistance and declining net condition (age, physical integrity) can further erode the expected protective effect of LLINs/ITNs in certain contexts [49]. Together, these considerations underscore the need to interpret associations between net use and LF in light of local vector ecology, temporal patterns of net use, and product performance.

Beyond vector bionomics, species-specific transmission ecology may further contribute to heterogeneity in observed associations. Indonesia is unique in harbouring three filarial parasites with distinct reservoir dynamics [11]. Zoonotic strains of *Brugia malayi* are reported predominantly in western Indonesia and may involve animal reservoirs, whereas *Brugia timori* and *Wuchereria bancrofti* are considered strictly anthropophilic [50]. These ecological differences imply that transmission persistence may not be driven solely by human behavioural or sanitation factors in all regions, particularly where zoonotic reservoirs sustain parasite circulation. Because SKI 2023 does not include species-level identification or information on animal reservoir proximity, our analysis could not stratify findings by parasite species or reservoir type. Consequently, associations observed in this nationally representative dataset should be interpreted within the context of potentially differing ecological systems across western and eastern Indonesia. Future investigations integrating parasitological confirmation, ecological stratification, and veterinary surveillance would allow more precise identification of species-specific risk patterns and further strengthen One Health–orientated elimination strategies.

The results of this study revealed a strong association between malaria/dengue co-infection and LF. Several studies have reported co-endemicity and potential co-transmission between mosquito-borne diseases, which may be related to immunological interactions and co-exposure [31], [51], [52], [53]. This phenomenon can be interpreted when the same mosquito species is capable of transmitting more than one pathogen—for example, some *Anopheles* species are capable of transmitting both *Wuchereria bancrofti* (which causes filariasis) and *Plasmodium* (malaria)—while some *Aedes* that play a role in sub-periodic filariasis (e.g., *Ae. polynesiensis*, *Ae. samoanus*) coexist with dengue-carrying *Aedes* species (e.g., *Ae. aegypti*, *Ae. albopictus*), resulting in overlapping risk in certain areas [31], [51], [52], [53]. This phenomenon was proven by several local studies in several regions in Indonesia, which showed that LF is also transmitted by malaria and dengue vectors such as *Anopheles* spp. and *Aedes* spp. [38], [54], [55]. In addition, the synchrony of biting behaviour and parasite periodicity, as well as local ecology that determines the presence and abundance of vector species, also play a role in this co-infection [51], [53]. Therefore, co-endemicity/coinfection requires integrated surveillance approaches and cross-disease vector control strategies in endemic areas [31], [51].

These findings support a One Health-style, multi-sector approach to LF elimination. Firstly, expanding directly observed treatment—for example, by training and deploying more community health volunteers or integrating MDA delivery with other programmes—can improve compliance and coverage. Indonesia's own programmes have emphasised volunteer treatment supervisors to ensure that every single person ingests the medicine. Scaling up such efforts, especially in urban and challenging-to-reach communities, should be a priority. Secondly, national and local programmes should coordinate malaria, dengue, and LF vector interventions through Integrated Vector Management (IVM). This could include mapping areas where *Aedes*-driven LF persists and applying control methods effective against day-biting mosquitoes (e.g., covering water containers, larviciding, and community cleanup) as well as reinforcing *Culex* measures (e.g., improving drainage and targeted residual spraying). Thirdly, WaSH improvements must be integrated into the LF strategy. Investments in sanitation—converting pit latrines to ventilated improved systems, preventing sewage stagnation, and promoting household hygiene—will reduce *Culex* breeding and confer broader health gains. Fourthly, and finally, integrated surveillance and coordination are essential. Regular entomological surveys (including xenomonitoring for filarial infection in mosquitoes) should be merged with malaria and dengue surveillance to identify hot spots of all three diseases. Health data systems can flag co-endemic areas for joint intervention. One Health coordination—involving human health, environmental sanitation, and even veterinary sectors (to monitor zoonotic *Brugia*)—will maximise the impact of limited resources and help Indonesia meet its 2030 elimination targets.

### 4.1 Limitations

This study has several limitations that should be considered when interpreting the findings. First, the analysis is a cross-sectional, secondary study of the 2023 Indonesia Health Survey (SKI 2023), which precludes inference of temporality or causality between exposures and LF outcomes. Secondly, the outcome measure—self-reported, clinically diagnosed LF presenting as lymphoedema or hydrocele—captures chronic, often irreversible sequelae of past infection rather than current parasitological status. Lymphoedema and hydrocele typically develop months to years after initial infection and can become clinically apparent only after adult worms have died or following treatment; therefore, clinical cases identified in the survey may reflect infections acquired long before the period covered by the measured programme or household exposures. This temporal discordance can decouple current behaviours or recent MDA participation from disease status and may attenuate or bias associations with contemporaneous variables. Thirdly, reliance on self-reported clinical diagnosis introduces potential misclassification (both false positives and false negatives) and recall or reporting biases; the survey did not include objective parasitological tests such as circulating filarial antigen (CFA) assays or microfilaria microscopy, nor molecular diagnostics or xenomonitoring in vectors that would indicate active or recent transmission. Fourthly, behavioural covariates (e.g., bed net, repellent, and coil use) are self-reported and therefore susceptible to recall and social desirability biases, which could account for some counterintuitive associations observed. Fifthly, residual confounding remains possible: although multivariable models adjusted for multiple individual-level covariates, unmeasured community-level factors (local MDA history and coverage, micro-environmental breeding sites, and insecticide resistance status) may influence observed relationships. Sixthly, and finally, despite the national representativeness of the dataset, sub-district and village heterogeneity may limit the direct applicability of national estimates for local programme decision-making. Future studies should incorporate objective parasitological and molecular diagnostics, paired entomological surveys, and longitudinal designs to better link exposure, recent infection, and subsequent clinical outcomes.

## 5 Conclusion

Based on a national analysis of the SKI 2023 dataset, this study found that LF was most frequently reported among children (0–17 years) and was slightly more common in females. Failure to receive directly observed preventive chemotherapy and the use of pit/open-hole latrines were associated with higher odds of LF, while co-endemicity with malaria and dengue fever indicates an ecologic overlap of vectors that is relevant for integrated control. These findings underscore the need to strengthen directly observed mass drug administration (MDA), integrate water, sanitation, and hygiene (WaSH) improvements with targeted vector management (addressing both diurnal and nocturnal vectors), and incorporate entomological surveillance and parasitological diagnostics to guide evidence-based prioritisation. Multisectoral, locally tailored One Health approaches are likely to accelerate progress toward national LF elimination targets.

## CRediT authorship contribution statement

**Yoerdy Agusmal Saputra:** Writing – review & editing, Writing – original draft, Visualization, Project administration, Methodology, Data curation, Conceptualization. **Rohana Uly Pradita Siregar:** Writing – review & editing, Writing – original draft, Visualization, Methodology, Formal analysis, Data curation.

## Ethics declaration

### Informed consent and patient details

Written informed consent to take part in the study and to publish the article has been obtained from all participants or their legal representatives. The privacy rights of participants have been observed.

### Studies in Human

This study was performed in compliance with relevant laws, regulatory frameworks and guidelines where the research took place.

This study was approved by the The Health Research Ethics Committee, Faculty of Public Health, Universitas Sriwijaya.

(Approval No. 680/UN9.FKM/TU.KKE/2025)

## Declaration of generative AI and AI-assisted technologies in the writing process

During the preparation of this work, the author(s) used ChatGPT to enhance language and readability. After using this tool/service, the author(s) reviewed and edited the content as needed and took full responsibility for the content of the published article.

## Funding

None.

## Declaration of competing interest

The authors declare that they have no competing interests associated with the material presented in this paper.

## Data Availability

The authors do not have permission to share data.
